# Accurate Image Analysis of the Retina Using Hessian Matrix and Binarisation of Thresholded Entropy with Application of Texture Mapping

**DOI:** 10.1371/journal.pone.0095943

**Published:** 2014-04-29

**Authors:** Xiaoxia Yin, Brian W-H Ng, Jing He, Yanchun Zhang, Derek Abbott

**Affiliations:** 1 Centre for Applied Informatics & College of Engineering and Science, Victoria University, Melbourne, Australia; 2 Centre for Biomedical Engineering (CBME) and School of Electrical & Electronic Engineering, The University of Adelaide, Adelaide, South Australia, Australia; University of Florida, United States of America

## Abstract

In this paper, we demonstrate a comprehensive method for segmenting the retinal vasculature in camera images of the fundus. This is of interest in the area of diagnostics for eye diseases that affect the blood vessels in the eye. In a departure from other state-of-the-art methods, vessels are first pre-grouped together with graph partitioning, using a spectral clustering technique based on morphological features. Local curvature is estimated over the whole image using eigenvalues of Hessian matrix in order to enhance the vessels, which appear as ridges in images of the retina. The result is combined with a binarized image, obtained using a threshold that maximizes entropy, to extract the retinal vessels from the background. Speckle type noise is reduced by applying a connectivity constraint on the extracted curvature based enhanced image. This constraint is varied over the image according to each region's predominant blood vessel size. The resultant image exhibits the central light reflex of retinal arteries and veins, which prevents the segmentation of whole vessels. To address this, the earlier entropy-based binarization technique is repeated on the original image, but crucially, with a different threshold to incorporate the central reflex vessels. The final segmentation is achieved by combining the segmented vessels with and without central light reflex. We carry out our approach on DRIVE and REVIEW, two publicly available collections of retinal images for research purposes. The obtained results are compared with state-of-the-art methods in the literature using metrics such as sensitivity (true positive rate), selectivity (false positive rate) and accuracy rates for the DRIVE images and measured vessel widths for the REVIEW images. Our approach out-performs the methods in the literature.

## Introduction

Retinal vascular disorders refer to a range of eye diseases that affect the blood vessels in the eye. Assessment of vascular characteristics plays an important role in various medical diagnoses, such as diabetes [1], [2], hypertension [3] and arteriosclerosis [4]. Retinal vessel segmentation algorithms are a fundamental component of computer aided retinal disease screening systems. Manual delineation of retinal blood vessels is a long and tedious task and requires extensive training and skill [5]. This motivates accurate machine-based quantification of retinal vessels that assist ophthalmologists in increasing the accuracy of their screening processes, allowing fewer highly trained individuals to carry out the screening processes, which may be of clinical benefit [6].

Fundus photography involves taking digital images of the back of the eye, such as the retina, optic disc, and macula [7]. Fundus photography is used clinically to diagnose and monitor progression of a disease. It is needed to obtain measurements of vessel width, colour, reflectivity, etc. State-of-the-art algorithms can be divided into a few main categories that deal with retinal vessel segmentation from fundus photographs, and recent review papers have already discussed these in some detail [8], [9]. We include only a brief summary of these reviews to sufficiently set the context for our work.

Classifier based approaches are perhaps the simplest. Two distinct categories of pattern classification techniques for vessel segmentation are supervised (which requires training) [10] and unsupervised (which do not) [11]. Training a classifier uses datasets of manually labelled vessel images to allow the classifier to recognise retinal vessel regions from the background; such techniques have been employed by Nekovei and Ying [12], Staale *et al.* [5] and Soares *et al.* [13], among others. In contrast, unsupervised classifiers attempt to find, directly, inherent differences between blood vessels and the background in images of the retina; examples include fuzzy C-means clustering [14] and Bayesian classification [15]. The finding in [8] is that in general, supervised classification has improved performance over unsupervised schemes, although the performance is affected by issues such as non-uniform illumination.

Apart from classifier-based approaches, other main categories of techniques in the literature include: matched filtering, morphological processing, vessel tracing, multi-scale processing, and model-based approaches [8]. Matched filtering attempts to find correlation between a fundus photograph with templates of vessels, known as kernels. Examples are reported in [16]–[18]. Regions with high correlation are thus detected as blood vessel structures. Another method, which uses matched filtering, is based on a binarisation via local entropy that is applied by Villalobos-Castaldi *et al.* [19] for vessel segmentation. The binarization approach involves a matched filter for vessel enhancement with combination of the gray-level co-occurrence matrix to calculate a statistical feature in relation to local entropy. The statistical feature acts as a threshold value for segmentation of the vessel network. Morphological processing applies operations with pre-determined structuring elements, designed to capture certain shapes, to the image. To carry out vessel segmentation with morphological operations, the base assumption is that vessels are constructed as connected linear segments. Such approaches possess an improved reduction in noise within the segmentation result, but the main disadvantage lies in a lack of ability to fit complex vessel structures. Examples of this approach in the literature can be found in [20], [21]. Vessel tracking attempts to find the path that best matches the vessels in a given image, subject to pre-determined vessel profile models, by following vessel centre lines using local characteristics for guidance. Such approaches [22]–[24] lead to accurate vessel width calculation and can identify individual vessel segments that other methods struggle to find. Multi-scale approaches exploit the fact that vessel widths decrease as they extend away from the optical disk. Many of the multi-scale algorithms involve vessel enhancement filters, such as in [25]–[27]. Model based approaches construct explicit models for vessels, designed to capture various properties, such as a Laplacian cross profile in intensity across a vessel [28], robust selection of blood vessel models [29] and deformable models [30], [31], for just a selection.

Studies in the literature provide inspiration for our proposed framework. In particular, our approach uses Hessian matrix analysis for curvature evaluation, which delineates the texture features of retinal vessels accurately, in combination with binarization via thresholded entropy to achieve a basic segmentation of retinal vessels. Fine-tuning the segmentation is performed by a further application of morphological operations to prune and identify the vessel segments and remove noise pixels. A novel aspect of this work is the separation of post processing for vessels of different thicknesses, partitioned into two broad classes. This is accomplished using texture mapping with a spectral clustering approach [32], and it assists in increasing the accuracy of final segmentation.

This paper makes three contributions. First, image enhancement is a significant pre-processing step in this paper's algorithm for smoothing retinal images and enhancing the contrast between vessels and the background. It aims to remove noisy regions from the overall retinal image to enable accurate segmentation of retinal vessels. One way to increase the image contrast is to enhance the image ridges associated with the retinal vessels. However, in order to better enhance vessels of different widths, traditional approaches require construction of multi-scale matched filters at multiple orientations [33]. In contrast, this paper's approach uses eigenvalue analysis of the Hessian matrix to enhance ridges in the retinal images without changing the filter width. The texture analysis using features from local area estimates then allows areas with predominantly different vessel widths to be discriminated from each other.

Second, accurate segmentation of retinal vessels has the potential to improve the diagnosis of retinal disorders. To achieve accurate segmentation, our approach pre-groups vessels using a morphology based spectral clustering technique. This produces a simple colour map of the fundus images according to textural vasculature features instead of complex threshold processing for the evaluation of multi-scale images. Thereafter, the connectivity constraint is applied to the extracted vessels, with the constraints varied according to different texture regions: for regions where fine-grained noise in relation to small vessels are dominant, a smaller connectivity constraint is selected, and vice versa for regions that mainly consist of coarse-grained noise in relation to large vessels. The texture mapping operation also yields a partition of the pathological vessels from heathy ones, and allows to accurate removal of noise via morphological processing, aiming for accurate isolation of noise from vessels. The results in this paper show that the proposed algorithm outperforms other supervised and unsupervised segmentation methods in achieving high accuracy.

Third, one of the key goals of this paper is to achieve mostly automatic width measurement of blood vessels in retinal images from the segmented vessels. An important step in measuring retinal vessels is to extract centrelines and localise vessel edges from the segment image, by making use of the thinning morphology operation and calculating the number of pixels with overlap between the line perpendicular to each of the local vessel centrelines and the pixels from vessel segments. In this method, we introduce a 3×3 window to deal with each of the vessel branches, which is especially effective for vessel branches that cross. Using images from the REVIEW database [34], we show that our algorithm is capable of achieving a high level of accuracy and low measurement error, both for low and high resolution images. The algorithm described here automates of the analysis of retinal vessel widths, and is capable of finding widths at all points along the length of each vessel rather than at specific points of interest.

## Materials and Methods

**Figure 1:**
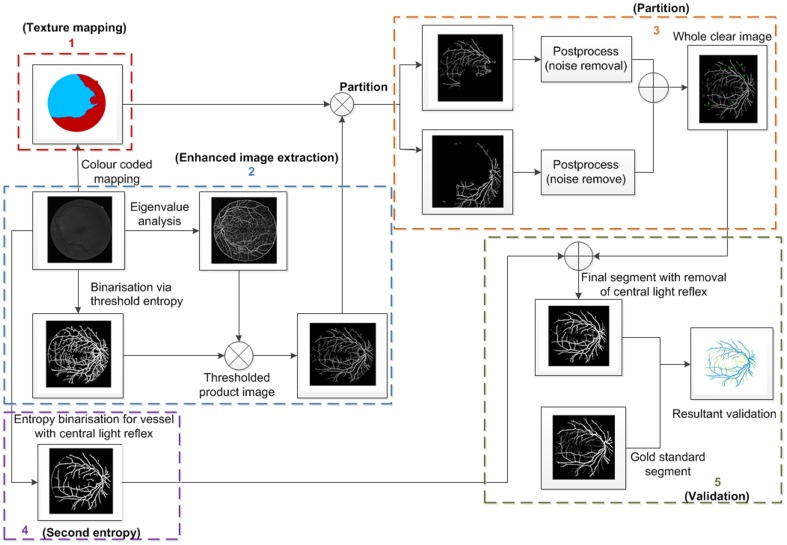
Illustration of the flow chart regarding the proposed retinal image segmentation algorithm. We number each of the steps in this figure from 1 to 5, which are associated with texture mapping, enhanced image extraction, partition, entropy binarisation for vessels with central light reflex (second entropy), and validation, respectively.

The method presented in this paper is based on unsupervised classification by finding inherent patterns of blood vessels in retinal images that can then be used to determine whether a particular pixel belongs to a vessel or not. The method uses region-based properties of retinal blood vessels for segmentation via using colour coded mapping to partition eigenvalue related enhancement of retinal images. A flow chart for image segmentation process is shown in Fig. 1; subsequent discussions of the details of our method will refer to steps illustrated in this figure.

### Image sources

The standard paradigm for validating medical image processing algorithms is to compare their outputs with a ground truth, or gold standard, generated by one or more human experts. To enable comparative assessment, we use image and associated manual segmentations from two public data sets available on the web, DRIVE [5] and REVIEW [35]. Both DRIVE and REVIEW databases include ground truth segmentations for their images.

The DRIVE database contains 40 colour images of the retina, 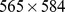 pixels per colour channel from three colour channels, represented in LZW compressed TIFF format. These images are originally captured from a Canon CR5 non-mydriatic 3 charge-coupled device (CCD) camera with a 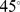 field of view (FOV), and are initially saved in JPEG-format. In addition to the colour images, the database includes binary images with results of manual segmentation. The 40 images are divided into a training set and a test set by the authors of the database. The results of the two manual segmentations are available for all the images of this test. The twenty colour images from a test set are to be analysed. One set of gold standard binary images and one set of manually segmented binary images showing blood vessels are made available. To validate blood vessel width measurements, we use the REVIEW database, because this database also offers gold standard vessel measurements. These images are of higher resolution than the DRIVE images, ranging in size from 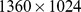 to 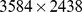 pixels. In all cases, the colour images are converted to grayscale by extracting the green channel information, because the green channel exhibits the best contrast for vessel detection [36]. To improve the local contrast of the retinal image, a preprocessing step, using morphological top-hat transform, is adopted from [37].

**Figure 2:**
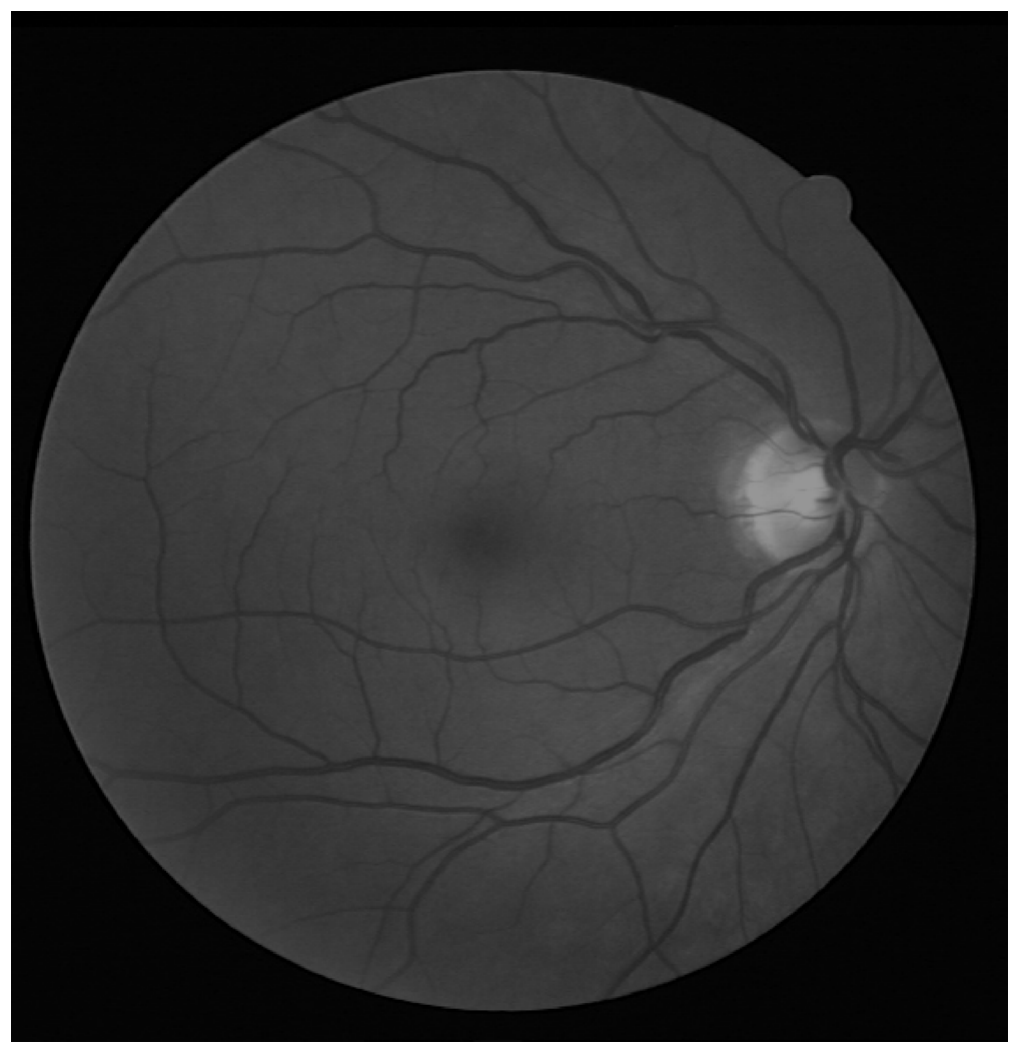
The green channel only image of a fundus photograph. The image is 02_test from DRIVE database.

Fig. 2 shows the green channel image that is selected from the original image named 02_test from the DRIVE database. The image is clear and shows no signs of any pathological tissues.

### Morphology Based Spectral Clustering

Photography of the eye fundus typically gives rise to complications such as inadequate contrast, lighting variations, influence of noise and anatomic variability affecting both the retinal background texture and the blood vessel structures [9]. Spectral clustering methods are promising approaches to perceptual retinal vessel segmentation that take into account global image properties as well as local spatial relationships. The method in [20], [38] integrates complex wavelet transforms with spectral clustering for a measure of spatial variation in texture via the morphological *watershed algorithm* [39]. It consists of four major stages. First, a dual-tree complex wavelet transform in the decimated domain is carried out to produce a set of image subbands. Next, a median filter is used to smooth the subband coefficients before the application of the gradient operator. The filtering operation is separable, scale- and orientation-adaptive, which produces nonlinear, edge-preserving smoothing and removes artificial noise from retinal images. The watershed algorithm, using image gradients, is then applied to the filtered image to produce an over segmented image. In our case, the implementation of the watershed algorithm [39] relies on the morphological H-minima transform, which controls over-segmentation. In the fourth stage, an image region similarity graph (RSG) is constructed from the over-segmented image. This is an undirected weighted graph where the set of nodes correspond to the atomic region (consisting of a set of connected pixels). For each pair of regions, the set of links represents relationships and the link weights represent similarity measures between the regions. Finally, we apply the spectral clustering technique to approximately solve this graph partitioning problem. This technique finds a partition of the graph such that the edges between different groups have a very low weight (which means that points in different clusters are dissimilar to each other) and the edges within a group have high weight (which means that points within the same cluster are similar to each other) [32].

**Figure 3:**
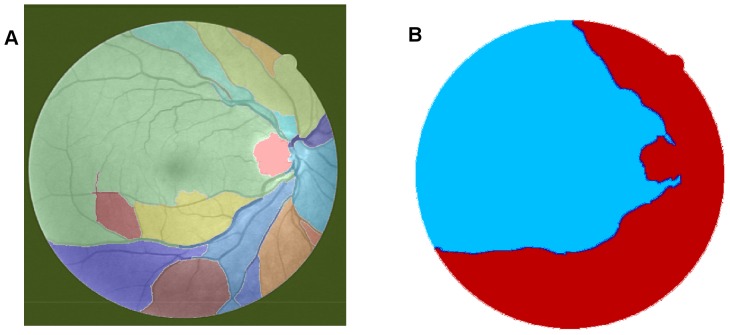
Illustration of the texture-based partitioning of fundus photograph. (A) Colour-coded mapping of the vessel texture, with the original image named 02_test from DRIVE database. (B) Colour-coded mapping of the two partitions of vessel texture: one is dominated by small blood vessels (labeled by blue colour) and the other is mainly controlled by large blood vessels (labeled by red colour).

In the context of this paper, spectral clustering groups together those regions in the RSG that have come from the same perceptual region, as illustrated in Fig. 3(A). This step produces the colour coded mapping contained in outline box 1 in Fig. 1. The texture features are grouped together according to a region's predominant blood vessel size, as demonstrated in Fig. 3(B). We then merge all the small regions (Fig. 3(A)) into two large regions (Fig. 3(B)) according to the local texture, i.e. fine-grained noise and coarse-grained noise. The detail regarding the texture will be discussed later in this manuscript. We manually adjust the threshold according to the connection limitation with application of morphology close operations. After application of different value of selected threshold to connection limitation, it is expected that the connection limitation with smaller threshold allows us to keep the small vessels as much as possible and filter out most fine-grained noise from background; for the connection limitation with larger threshold, it is expected to filter out most coarse-grained noise and obtain larger vessel branchings as clear as possible. An threshold is selected randomly and then we adjust it to see if there shows obvious change in texture of noise. If it exists, the spectral cluster can be used to regroup these small clusters.

### Eigenvalue Analysis of Hessian Matrix

**Figure 4:**
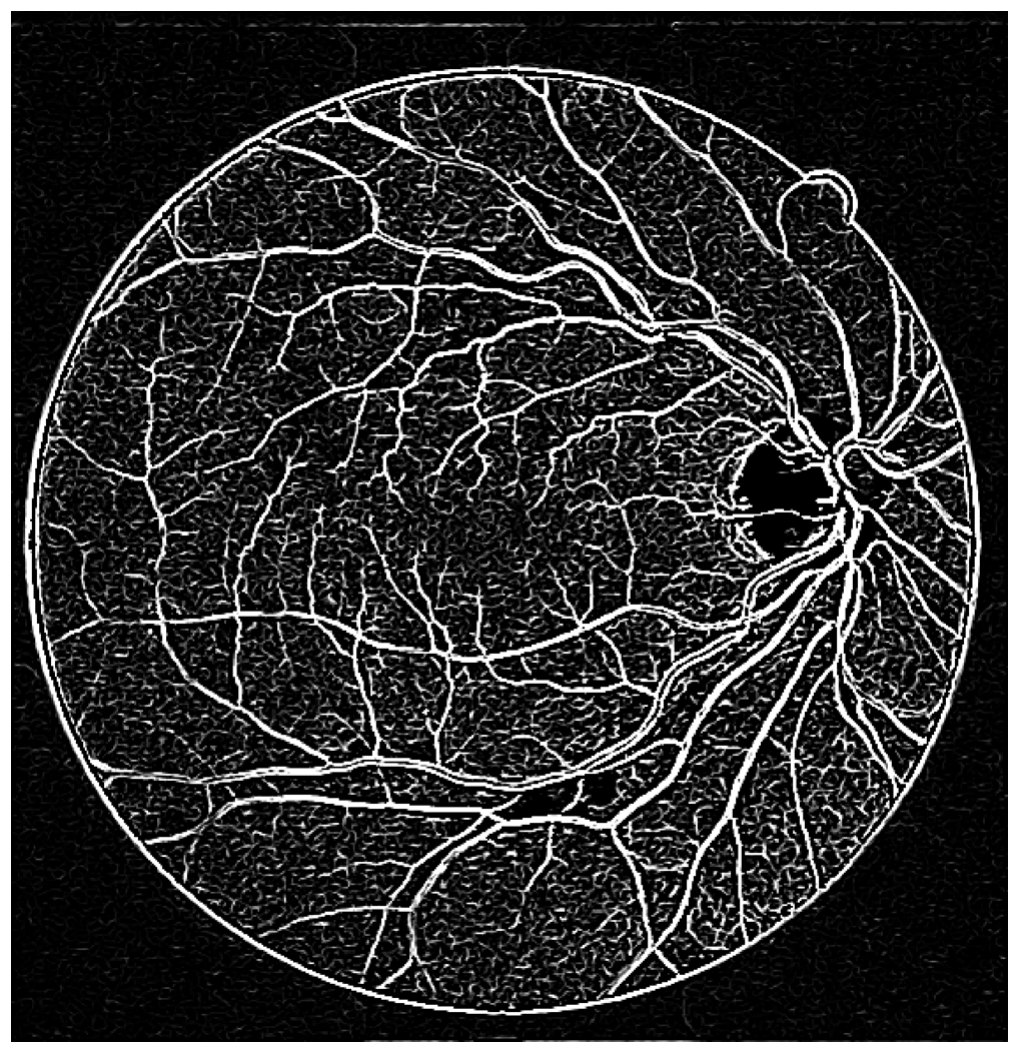
Illustration of a curvature based enhancement of the image of the retina via the eigenvalue analysis of Hessian matrix. The image used is 02_test from the DRIVE database.

The vessel enhancement technique used in this paper is an eigenvalue analysis of the image Hessian matrix at a single scale, and is adapted from the multiscale version of Frangi *el al.* [25]. The fundus photograph is once again pre-processed using the top-hat transformation to produce the image 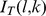. The local behaviour of the pre-processed image 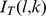 can be determined from its second order Taylor's series expansion in the neighbourhood of a point 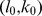. The idea behind eigenvalue analysis of the Hessian 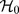 is to extract the principal directions in which the local second order structure of the image can be decomposed [25]. In this case, the direction of smallest curvature along the vessel can be computed directly. This is achieved by finding the eigenvectors corresponding to the smallest eigenvalues. Fig. 4 shows the enhancement via eigenvalue analysis.

### Maximum Entropy Binarisation

When a grayscale image is binarised, a threshold value must be specified. In our approach, the optimum threshold value is determined as the pixel intensity from the histogram of the image that exhibits the maximum entropy over the entire image. To represent spatial structural information of an image, a co-occurrence matrix is generated from the pre-processed image. It is a mapping of the pixel to pixel greyscale transitions (i.e. the gray level 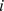 follows the gray level 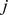) in the image between the neighbouring pixel to the right and below each pixel in the image. The co-occurrence matrix of the pre-processed image 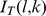 (element wise), satisfying with the equation 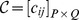, is a two dimensional matrix of size 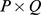, where the elements 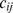 are defined as:
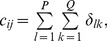
(1)where 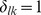, if
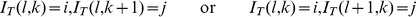
(2)


and otherwise, 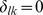.

The probability of co-occurrence satisfies the equation, 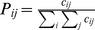. A threshold 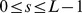 that divides an image into two classes, background and object, also divide the co-occurrence matrix into four regions representing within object (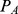), within background (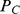), object to background (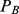), and background to object class transitions (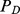). 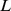 is the maximum intensity value of the image to be analysed. The second-order entropy of the object (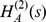) and background (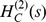) are defined as:
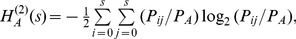
(3)




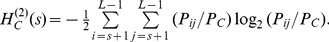
(4)


Both 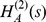 and 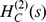 are functions of 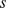. By summing up the local transition entropies, the total second-order local entropy of the object and the background is given by
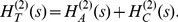
(5)


Finally, the optimal threshold 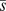 corresponding to the maximum of entropies 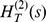 over 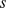 gives the optimal threshold for value [40]

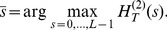
(6)


The final segmented binary mask of the vessel image is obtained by thresholding the pre-processed image 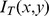 with the optimal threshold 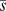:
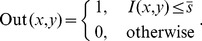
(7)


**Figure 5:**
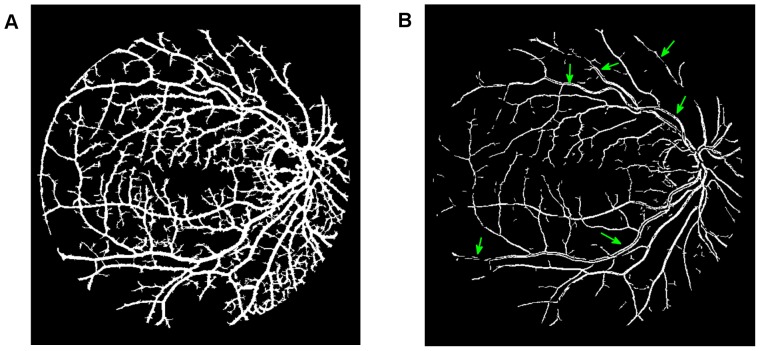
Multiplication of images with the original image named 02_test from the DRIVE database. (A) Illustration of the resultant mask used for extraction of the enhanced retinal vessels via entropy based binarisation. (B) A global thresholded image after combining (A) and Fig. 4.

In order to obtain the initial mask of retinal vessels, we select a smaller magnitude of the threshold at vessel pixels near the vessel edges. Finally, we multiply the eigenvalue based enhanced image (after threshold) shown in Fig. 4 with the entropy based mask shown in Fig. 5(A). The resultant image is shown in Fig. 5(B). The method performs well in extracting the enhanced retinal vessels from the background with significantly reduced noise compared to other unsupervised mask or segmentation techniques.

### Combining multiple segmentations to handle non-uniform illumination

**Figure 6:**
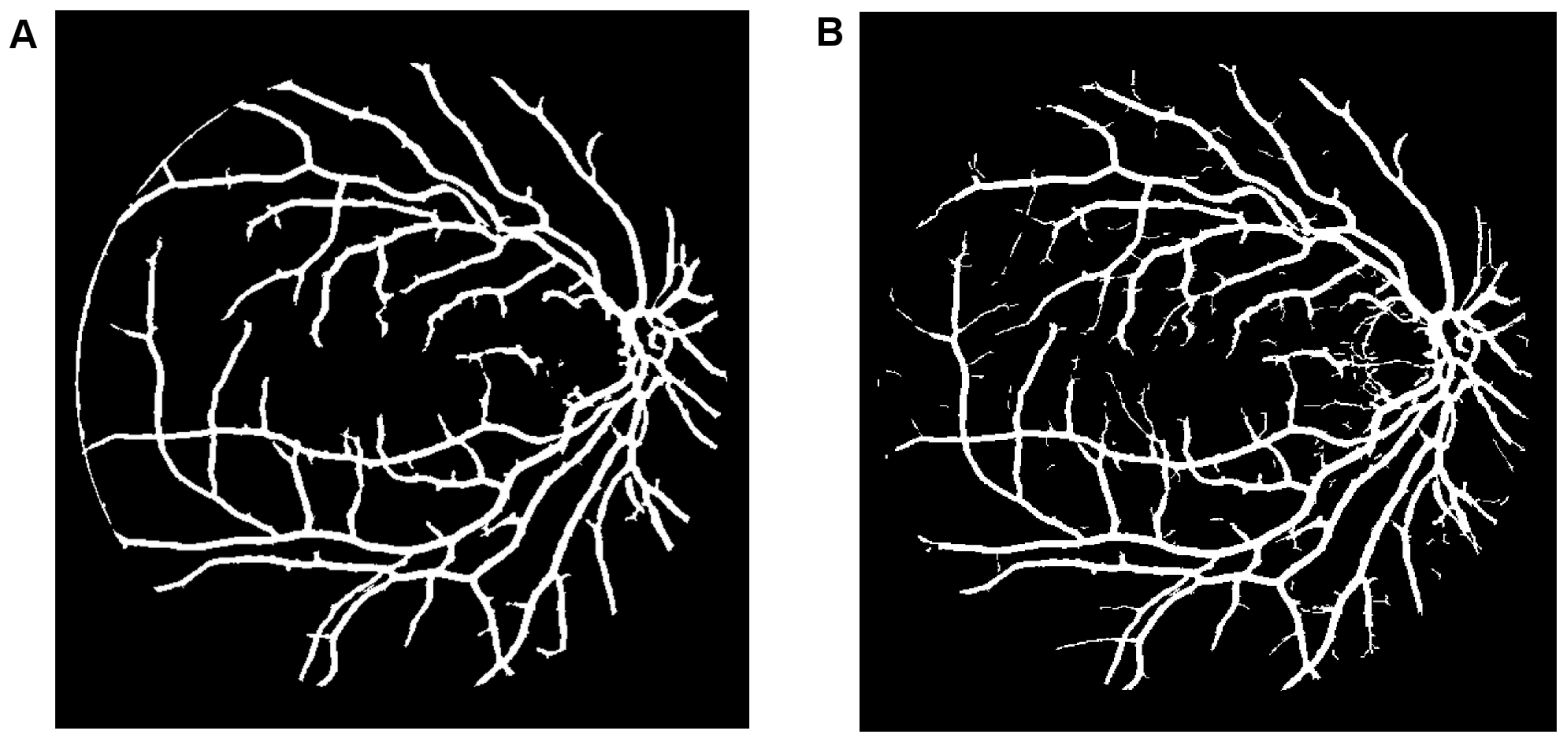
Outputs of interim processing steps. (A) Illustration of binarisation with threshold selected to maximise entropy. (B) Illustration of the final segmentation, where the effect of central light reflex, indicated by green arrows in Fig. 5(B) has been removed in the resultant image.

This subsection addresses accurate segmentation techniques when combining the applications of several classical image processing algorithms mentioned above. Segmentations using a curvature based method (Eigenvalue analysis) show obvious signs of central light reflex. According to Spencer [41], the normal light reflex of the retinal vasculature is formed by reflection from the interface between the blood column and vessel wall, and thicker vessel walls cause the light reflex to be more diffuse and have lower intensity [42], [43]. In order to eliminate the effect of the central light reflex, we repeat the binarisation procedure on the top-hat preprocessed images, but with a larger threshold at vessel pixels near the related centreline of the retina vessels affected by the central light reflex. We manually select the thresholds and calculate the ideal segments of the central light reflex vessels. The final segmentation is the superposition of the segmentation from the extracted enhanced image, as shown in Fig. 5(B) and binarisation via entropy shown Fig. 6 (A), where the effect of the central light reflex, indicated by green arrows in Fig. 5(B), has been removed in the resultant image, as shown in Fig. 6 (B). We name this the dual-threshold entropy approach, to position it in the overall taxonomy of retina vessel segmentation methods, e.g. [8].

**Figure 7:**
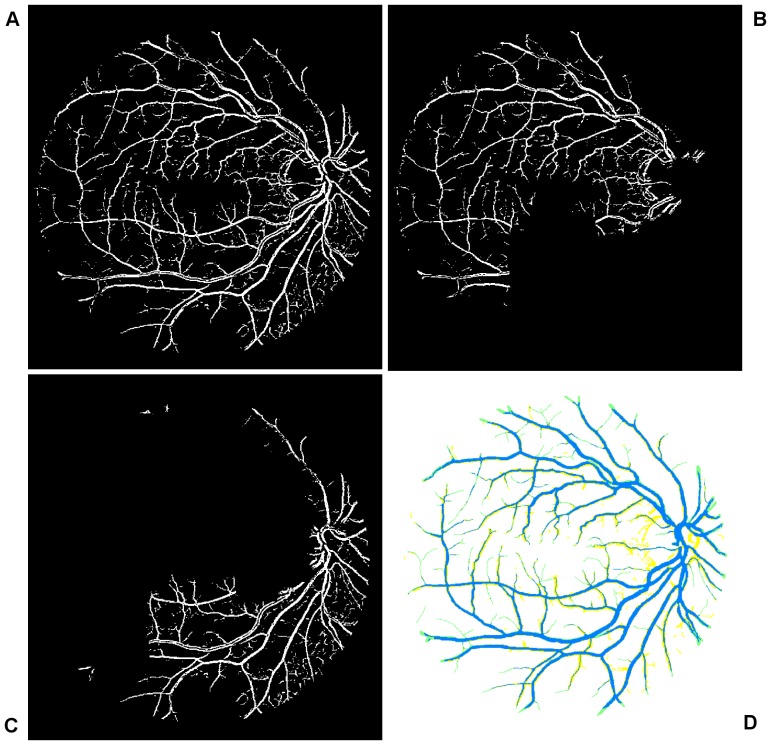
Overview of the main steps taken by our algorithm when processing a fundus image. (A) Illustration of globally thresholded image after multiplication between Fig. 4 and Fig. 5(A). (B) and (C) Illustration of two partitions of segmentation of (A) according to color coded mapping in Fig. 3(B). (D) Illustration of good overlapping (blue) between the resultant segment (yellow) and gold standard segment (green).

To achieve clear segmentation of blood vessels in the images of the retina, we conduct simple morphology post-processing, i.e. morphological connectivity constraint operations on the extracted curvature based enhanced images. The connectivity constraint is varied according to different background texture of noise that dominates the image. The fine-grained noise texture (small contiguous bright region) determines small connectivity constraint, and vice versa for coarse-grained noise texture (relatively large contiguous bright region). The morphological spectral clustering is applied for the identification of textural regions. This is to consider the fact that texture appearance is changing with image recording parameters, for instance, illumination variation and direction of view, a problem common to any real surface. The extracted segmentation of texture works like windowing an image, which determines window size, position and shape with different texture appearance, different intensity distribution associated with different texture of background noise [44]. Regarding non-uniformed illumination of a retinal image, it is normally partitioned into two regions obviously according to the variation of illumination with change of vessel size. For instance, the extracted enhanced image illustrated in Fig. 7(A), consists of two regions: for regions where fine-grained noise (in relation to small retinal vessels) are dominant, shown in Fig. 7(B), it is reasonable to select the smaller connectivity constraint than the regions that consist of coarse-grained noise related to large vessels, shown in Fig. 7(C). The vessel segments corresponding to different background textures are linearly combined to produce the whole segmentation for the curvature based enhanced image. A similar method is used for a retinal image with pathological tissue, which will be differentiated into two parts with and without the pathology. The criteria to merge these segments into two are as follows. If the illumination is non-uniform, the segment with noise can be divided into two parts: (i) a segment with fine-grained noise texture and (ii) a segment with coarse-grained noise texture. The texture of noise is associated with the size of much smaller contiguous bright regions from the background after segmentation, which is different from retinal vessels that contain larger contiguous regions of bright pixels. The two portions in relation to two different textures of noise lead to two different segments. For pathological tissue, we consider the texture segments that contain the pathological tissue. Therefore we locate two groups of segments: (i) the group of segments containing pathological tissues and (ii) the group of segments containing healthy tissue. This is illustrated in the subsection on *qualitative segmentation results*.

In order to achieve accurate segmentation of the retinal images, there are nine parameters produced in the segmentation procedure that need be adjusted manually. Actually, according to the resultant segmentation, it is found that such adjustments are simple and slightly changed among each retinal image. For reproducibility, these parameters are illustrated in the *discussion* section.

### Width Measurement

We propose a vessel width measurement method to identify a pair of edge points representing the width of a vessel at a specific center point. The first step is to apply a morphological thinning algorithm [11] on the segmentation to locate the centreline; thinning iteratively removes exterior pixels from the detected vessels, finally resulting in a new binary image containing connected line segmentation of “on” pixels running along the vessel centres. Thereafter, we apply a skeletonisation operation on the thinned vessel segments to detect the vessel centrelines. Skeletonisation is a binary morphological operation that removes pixels on the boundaries of objects without destroying the connectivity in an eight-connected scheme [45]. The remaining pixels make up the image skeleton without affecting the general shape of the pattern. Therefore, the one pixel thin vessel centreline is obtained with a recognizable pattern of the vessel. The pixels that consist of vessel centreline are viewed as a series of specific centre points for the subsequent width measurements.

**Figure 8:**
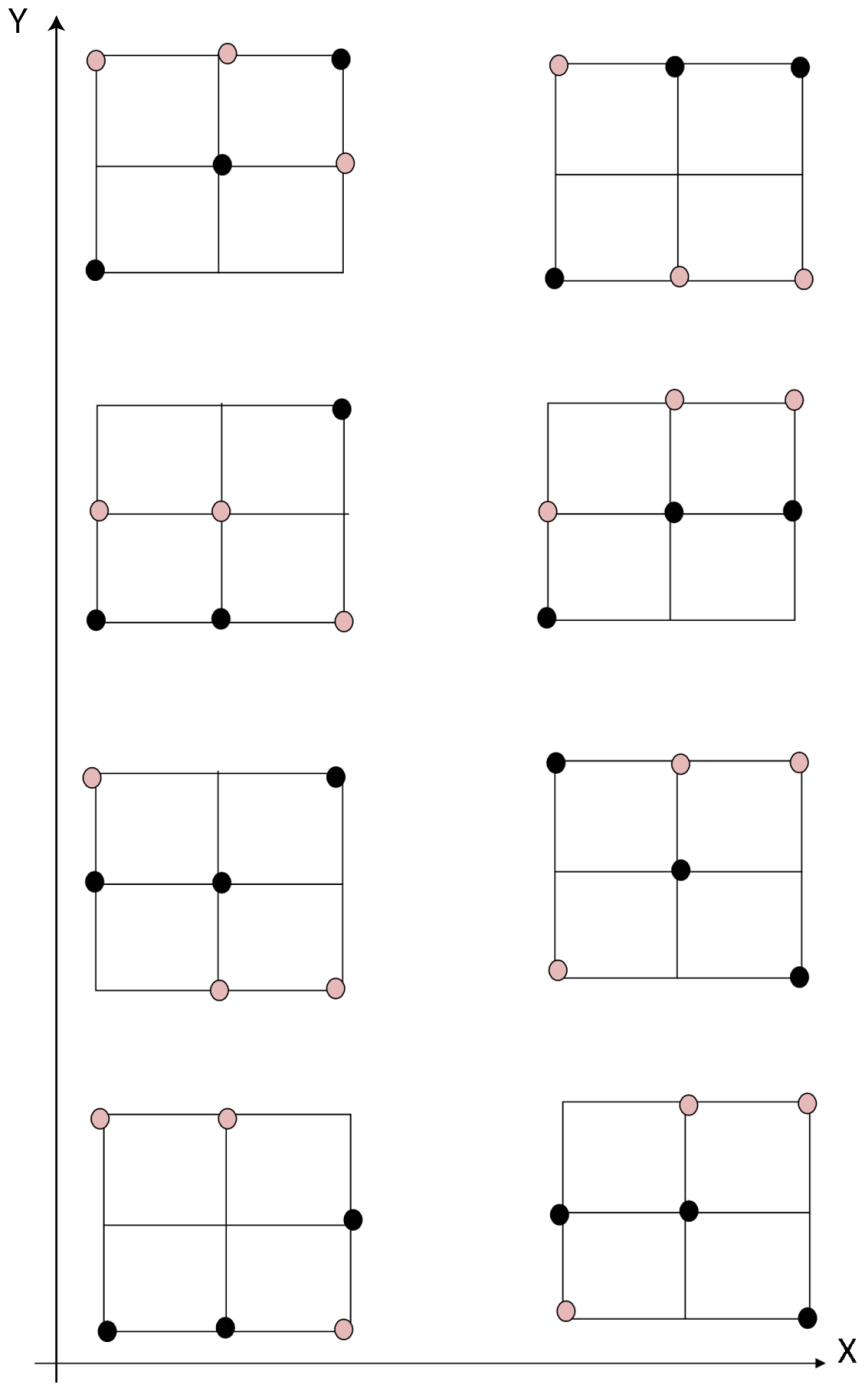
Represents 14 possible windows with three unique orientations along its horizontal axis. These 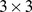 windows are used to detect all edge points on the vessel centreline image. These windows are convolved with the vessel centreline image. The pixels inside each window are the connected pixels consisting of only three unique coordinates, or along the 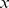-axis, or along the 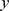-axis. We use black and pink dots to separately represent the possible positions of pixels involved in the window.

**Figure 9:**
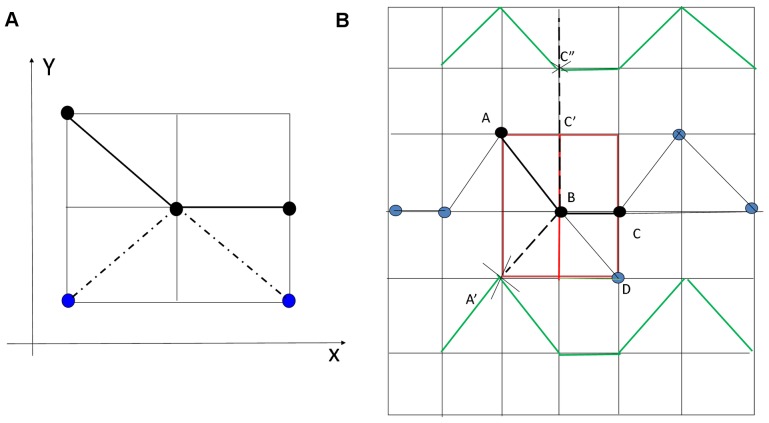
A schematic drawing that illustrates the processing steps of the width measurements. (A) Illustration of the pixels with black color used for edge detection and with the blue pixels to be viewed as branch pixels that are rejected for edge detection. (B) Illustration of the process of width measurement, which is used to determine the detected edge points.

All edge points are detected using 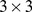 windows on the vessel centreline image using the following steps. First, we convolve the vessel centreline image with the window for the selected candidate points to be processed. We consider only three windowed centreline pixels, so that the positions of the three pixels along horizontal (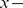) or vertical (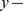) orientations are not repeated. Such windowed centreline pixels are aligned along one of 14 different possible orientations, illustrated in Fig. 8. Such aligned pixels as candidate pixels avoid vessel crossing to be detected with two adjacent branchings on the vessel centreline image. As shown in Fig. 9(A), the image pixels covered by the window consist of blue pixels and black pixels. The black pixels are validated as candidate pixels and the corresponding filter orientations along 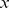 or 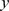 axis are regarded unique. Considering there are two groups of filter orientations, (consisting of the dash-dot line and the solid line with three centreline pixels, respectively), we select the pixels with larger 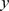 coordinates (black pixels) as candidate points for edge detection and the pixels with smaller 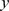 coordinates are rejected.

**Figure 10:**
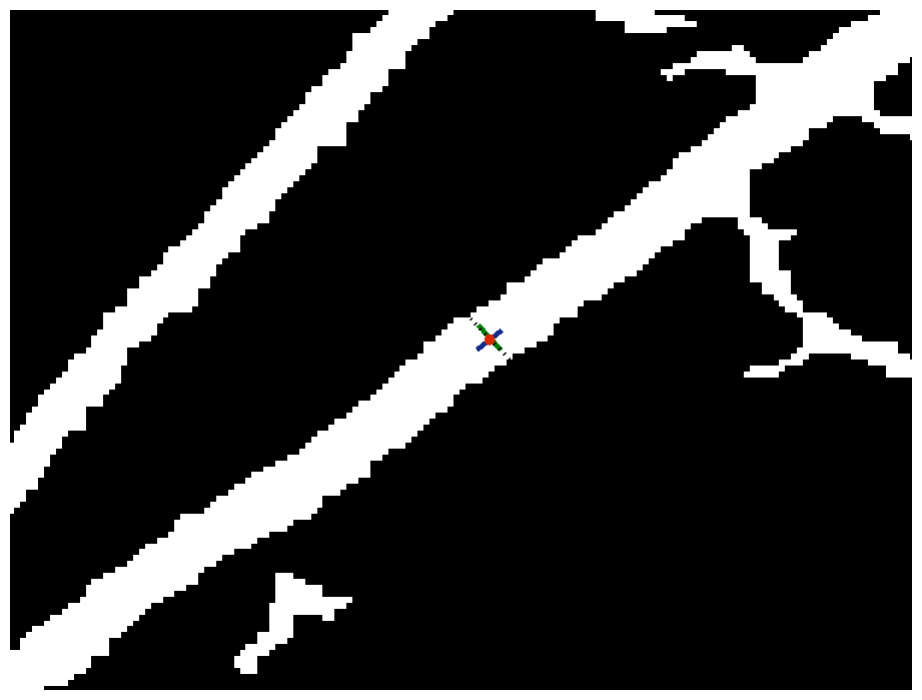
Illustration of width measurement via the retinal image segment. The blue centreline is rotated 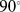 counterclockwise around the red central point, to the green solid line, overlapping with some of the white segment pixels.

We linearly extrapolate the pixels which form the centreline and make rotation afterwards, as a result, each of the resultant profile contains widest segmented pixels and pixels from additional background region. The principle is to approximate the tangent of windowed centreline mentioned above at any point of it via the connected neighbor pixels in the local region. The resultant profiles perpendicularly cross through tangents and go through the pixel with central coordinate. For windowed centreline consisting of pixels with the same 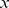 position, we directly find its perpendicular line. Such a resultant profile overlaps with vessel segment and its background, and the distances between the central coordinate pixels and the pixels from vessel segments are calculated. The edges of the extracted segments are located with the largest distances from the central coordinate points (centreline pixels). Fig. 9(B) illustrates such a process. In this figure, green solid lines indicate the observed retinal vessel edges. The line passing through the blue and black dots indicates the centreline. The thin black line BD is a vessel branch that should not be involved for edge detection. The black dots inside the red 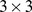 window form the centreline ABC. After a counterclockwise rotation of 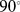 around the central point 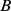, the line segment ABC maps to 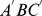. The extended line 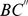 is the linear extrapolation of line 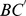 until it reaches the blood vessel edge. The points 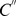 and 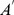 that are highlighted by the crosshairs are the intersection points between the blood vessels and line 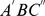. These two points are the detected edge points and the Euclidean distance between the two points is registered as the vessel width. Fig. 10 is an illustration of the centreline (in blue) that is rotated 90 degree counterclockwise around the central point (red), to the green solid line. The black dash line is the resultant positions of the candidate centreline pixels after rotation and exploration. The black dash line is overlapped with some of the white segment pixels. The length of these overlapped pixels is the measured vessel width for the corresponding red centre point.

## Results

The algorithm has been implemented in MATLAB version R2013a on a personal computer running Windows 7 with an Intel(R) Core(TM) i5-3470 CPU (3.20 GHz) and 8 GB of memory. On this platform, it takes about 24 seconds to process a DRIVE retina image to complete the segmentation. Considering that these results are obtained with MATLAB on a standard PC, the processing times are reasonable, and there is more headroom for improvement with further optimisation or customised hardware [46]. Even without speed improvements, our method can reasonably be incorporated into assisted-diagnosis systems and supply a result within an appropriate time frame (e.g. compared to a manual evaluation).

In the remainder of this section we first report qualitative results aimed at giving a visual appreciation for the quality of the vessel segmentation and vessel width measurements generated by our method. We then report the quantitative results for our method. The resultant vessel segmentation is calculated for images on the DRIVE database and compared with those reported by Jiang *et al.* [17], Perez *et al.* [27], and Zana *et al.* [20], Staal *et al.* [5] as well as human observation. The first three use unsupervised learning algorithms, and the last uses a supervised learning based algorithm. All these resultant segments are downloaded from the DRIVE database's website [47]. Typical vessel width measurements are performed on the REVIEW database. We compare the performance of our algorithm with the performance of two human experts. All these detected vessel edges are downloaded from the website containing the REVIEW database [34].

### Qualitative segmentation results

To evaluate the performance of segmentation, we apply our approach to all 20 images of the test set of the DRIVE database. Considering that the masks depicting the FOV included with the DRIVE images are not enough to clean the noise edge of the FOV produced by applying algorithms (shown in Fig. 4 and Fig. 5), for our segmentation implementation, we used a FOV mask computed simply by Sobel edge detection before applying a morphological closing operation. The use of the Sobel operator is to mark features on each side of a wide ridge and the closing operation close regions where multiple detected edges of blood vessels are close together. The morphological closing operation is conducted via a line shaped structural element with length of 3 at 12 directions.

To begin, we apply our method to process the image named 02_test from DRIVE database for illustrative purposes, as the retinal image is clear and without complicating pathology requiring further processing. Following our proposed method, top-hat based morphology preprocessing is first applied on the selected channel shown in Fig. 2 for contrast enhancement. Morphology based spectral clustering is then carried out in order to partition the fundus region, as shown in two colour-coded regions, as shown in Fig. 3. One forms a larger texture region which includes most of the smaller blood vessels (blue region); the other mainly consists of major vessels (red). Afterwards, eigenvalue analysis of Hessian matrix is conducted for an enhanced image. This is illustrated in Fig. 4. To extract the blood vessels from the background, entropy-maximising binarization is applied to yield Fig. 5(A). We then perform an element-by-element multiplication of Fig. 4 and Fig. 5(A), followed by a global thresholding, to obtain an initial segmentation, as illustrated in Fig. 7(A). Using the earlier two-colour partition, the segmentation is separated into Fig. 7(B) and Fig. 7(C), which correspond to the blue and red regions in Fig. 2, respectively. At this stage, the images are likely to be over-segmented, where many non-vessel pixels have been misclassified as vessels. However, the majority of the vasculature is represented by one large connected structure in the binary image, whereas misclassified pixels tend to be clustered to form isolated objects. It is not difficult to see that there is relatively larger connected structure in Fig. 7(C) than Fig. 7(B). Even the connectivity of misclassified pixels associated with isolated objects is also larger in Fig. 7(C) than the connected structure in related to small blood vessels.

By applying different connectivity constraints to the two sub-segmentations, we extract the curvature based segmentation, illustrated in Fig. 5(B), from the background. The clear segmentation is illustrated in Fig. 6(B). It is clear that a pronounced dark region runs through some of the vessels, indicated by green arrows. Then a second binarization is performed, with a larger threshold value to eliminate the effect of central light reflex, to produce the image in Fig. 6(A), where there are no dark regions going through of large pixels. To evaluate the retinal segmentation, we overlay the segmentation generated according to our method with gold standard segmentation. In Fig. 7(D), the blue colour indicates the overlapping pixels between the two segmentation, the yellow colour indicates the pixels found to be vessels by our proposed algorithm (this means they are false positives) and the green colour indicates vessel pixels in the gold standard segmentation (this means they are false negatives). The Fig. 7(D) shows good overlapping between the two segmentations with slight errors.

**Figure 11:**
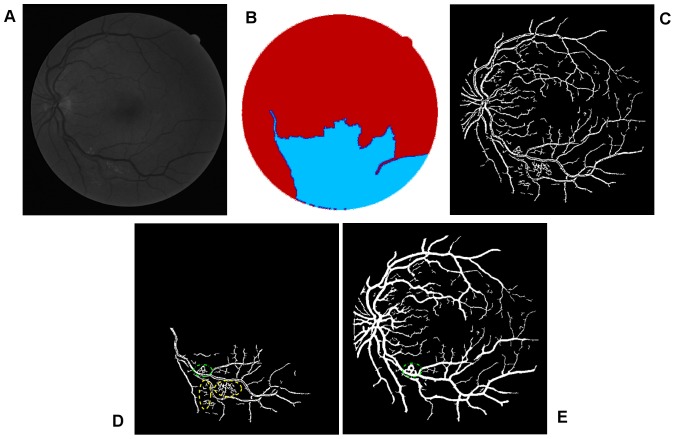
An example showing noise effects from a pathological image. (A) The green channel of original image named 03_test. (B) Colour coded mapping. (C) The extracted enhanced image after a global threshold and a global connectivity constraint. (D) The part in relation to blue coded mapping with pathological regions indicated by yellow dash and green dash-dot lines. (E) Final segmentation with part of pathological tissue existing indicated by green dash-dot line.

**Figure 12:**
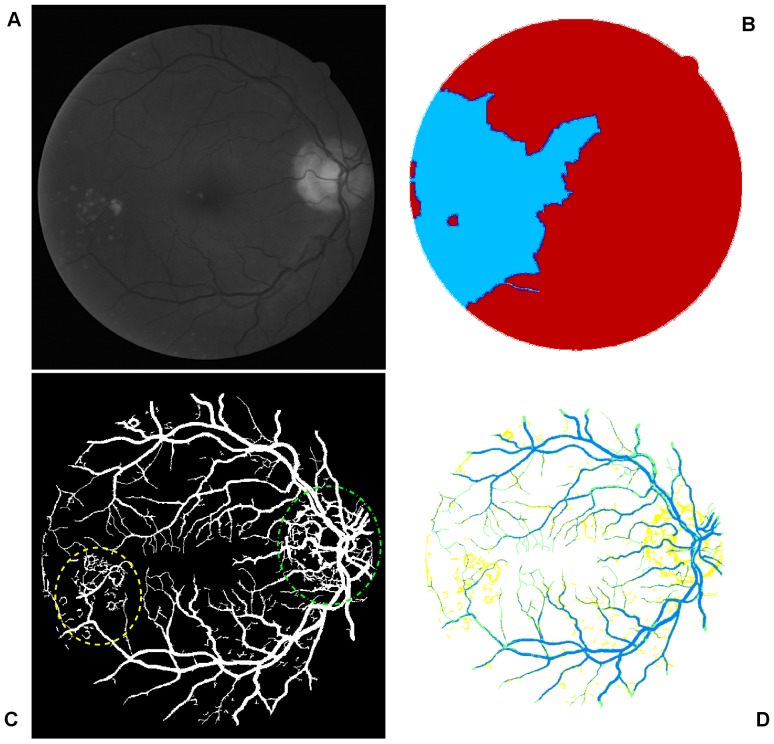
A further example showing the source of noise effects. (A) The green channel of original image named 08_test. (B) Colour coded mapping. (C) Final segmentation with noise effect partly from pathological tissue, indicated by yellow dash line, and partly from optic disk, indicated by green dash-dot line. (D) The superposition of the segmentation produced by our algorithm and manual segmentation, the yellow part of which represents the misclassified pixels of retinal blood vessels.

Fig. 11 and Fig. 12 show the effect of images containing pathological tissues, corresponding to images 03_test and 08_test from DRIVE database, respectively. Their green channel-only images are shown in Fig. 11(A) and Fig. 12(A). The extracted enhanced image associated with the original image of 03_test after a global threshold and a global connectivity constraint is shown in Fig. 11(C). It has been split into two parts according to the colour coded mapping shown in Fig. 11(B). The part in relation to blue coded mapping is shown in Fig. 11(D) is a region consisting of obvious pathological tissues, highlighted by yellow dash and green dash-dot lines. The diseased tissues circled by yellow dash line can be removed via specific connectivity constraint but leaving the potion circled by green dash-dot line that could not be removed, illustrated in Fig. 11(E). The binarisation via thresholded entropy used to diminish the central light reflex tends to broaden vessel size compared to the vessel size from extracted enhanced image. It leads to an increment of the noise effect. The pathological region that could not be removed thoroughly is one of reasons which lead to false identification of blood vessels. This then leads to an increased false positive rate of the resultant segment, which will be further explained and discussed in the quantitative analysis subsection.

Fig. 12(C) with the yellow dash-line further illustrates such a noise effect from pathological tissue. Though the coloured mapping shown in Fig. 12(B) has recognized the pathological tissue region, the tight connectivity between the diseased tissue and retinal vessels results in difficulty in the separation of pathological region. The region highlighted by green dash-dot line shows the noise from optical disk. We apply the red channel image to detect the optical disk region with morphology operation to remove the noise effect, but only part of the noise is removed, which is another reason leading to false segmentation of blood vessel. The comparison with manual segmentation is illustrated in Fig. 12(D). The segmentation with yellow coded is the false identification of blood vessels, most of which is concentrated in the pathological and optical disk region.

**Figure 13:**
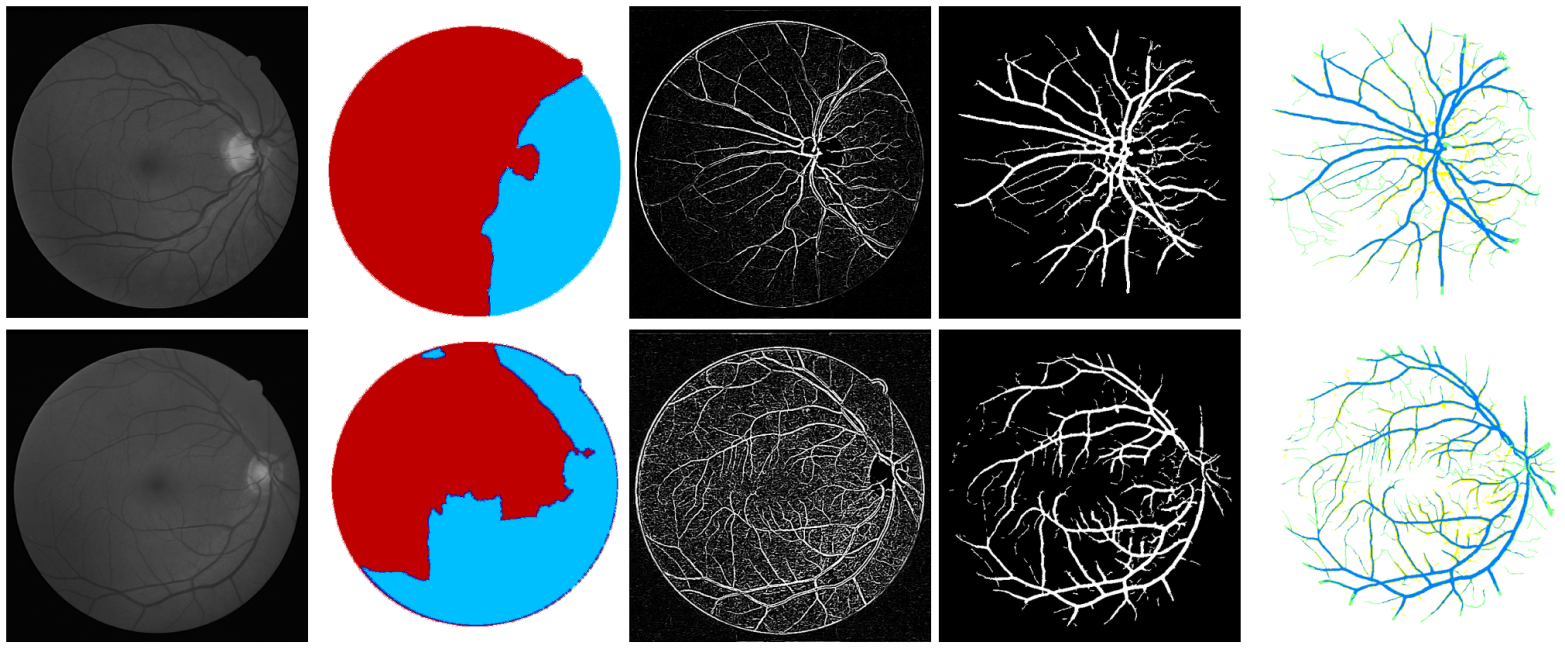
Overview of the main steps taken by our algorithm when processing more fundus images. From left to right, they are the green channels of the original images named 04_test and 13_test from DRIVE database, colour-coded mapping of the two partitions of vessel texture, results of enhanced images via eigenvalue analysis, masks using binarisation via thresholded entropy with difference size of noise corresponding to the coloured mapped partitions, final clear segmentation with remove of central light reflex, superposition of segments between the gold standard for retinal segmentation and the segmentation produced by the proposed algorithm.

More results of the proposed method that are related to the difference size of noise corresponding to the coloured mapped partitions, as applied to images 04_test and 14_test in the DRIVE database, are illustrated in Fig. 13.

### Quantitative segmentation results

Three measures are used to quantitatively assess our algorithm's performance: true positive rate (TPR), false positive rate (FPR) and accuracy (ACC). Note that TP and TN are the number of blood vessel pixels and background pixels which are correctly detected, respectively; FP is the number of pixels not belonging to a vessel, but is recognised as one, and FN is the number of pixels belonging to a vessel, but is recognised as background pixels, mistakenly. Based on these definitions, TPR, FPR and ACC are defined as follows:
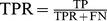
(8)




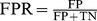
(9)




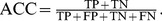
(10)


The three measures are calculated for the final segmentations from our algorithm, as well as several other retina vessel segmentation algorithms in the literature. We divide the 20 test images from DRIVE database into three classes: the images with approximately uniform illumination throughout the background and well localised central light reflex (Class 1); the images with approximately uniform illumination but with broken vessels due to central light reflex effect (Class 2); the images with non-uniform illumination and pathological tissues (Class 3). Characteristics of these three class members are easily observed. According to different classes of the images, we adopt different steps of our algorithm. For example, well localised central light reflex is viewed as the large vessels with thin central light reflex effect compared to the space between the vessels and their neighbours. The uniform illumination is viewed such that after a global threshold, the size of background pixels is distributed uniformly, and are not obviously large in one region and small in another. The pathological tissues in retinal images show complex morphology, appearing as a bright protuberance on or around a vessel branch. For the first class of the images with enough spacing between two vessels, we apply multiplication between curvature segmentation and binarisation, with morphological closing operation to eliminate the central light reflex. We adopt disk-shaped structuring element with the radius of 2 pixels to conduct morphological closing operation. For the second class of the images, where the spacing is small and the large vessels show low contrast, we apply entropy filtering to eliminate the central light reflex, after multiplication operation for segmentation. For the third class of the images, we apply the entire set of operations of our algorithm. In Table 1, we list the steps involved to process the three classes of retina images.

**Table 1:** The steps are involved to process three class members of retina images.

Class member	Class 1	Class 2	Class 3
Steps numbered (shown in Fig. 1)	2+5	2+3+5	1–5

The numbered steps are illustrated in Fig. 1.

The average quantitative results of the three classes of images are listed in Tables 2–4, while the average results of the overall set of 20 images are listed in Table 5. The quantitative performance of our method with the other approaches in terms of TPR, FPR, and ACC is compared as well as the percentage of improvement (Imp) between our method (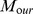) and the methods represented in literature (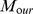). The percentage of improvement satisfies the equation: 
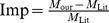
. The hand segmented images from the first manual observer are used as the benchmark. True and false positive rates (TPR and FPR) are included where these are available in the DRIVE database web site. Improving on the accuracy score of the second observer is not necessarily beneficial, since the choice of the first observer as the benchmark is arbitrary [11].

**Table 2:** Quantitative evaluation of vessel segmentation algorithms related to the first class of the images.

Method	TPR	FPR	ACC	Improvement ( 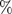 )	TPR	FPR	ACC
Our method	0.6988	0.0267	0.9504				
Jiang *et al.* [17]	0.5993	0.031	0.9238	our method vs Jiang *et al.*	17	−0.14	2.9
Perez *et al.* [27]	0.5772	0.0367	0.9172	our method vs Perez *et al.*	21.1	−27.2	3.6
Staal *et al.* [5]	0.674	0.0178	0.9566	our method vs Staal *et al.*	3.7	0.5	−0.6
Zana *et al.* [20]	0.6287	0.0197	0.9375	our method vs Zana *et al.*	11.1	35.53	1.4
2nd observer	0.7825	0.0378	0.9460	our method vs 2nd observer	−10.7	−29.37	0.5

Comparison of performance between the recent studies according to the first class of the images, including 6th, 9th, 12th, 17th, 18th, 20th test images from the DRIVE database.

**Table 3:** Quantitative evaluation of vessel segmentation algorithms related to the second class of the images.

Method	TPR	FPR	ACC	Improvement ( 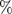 )	TPR	FPR	ACC
Our method	0.8045	0.0416	0.9444				
Jiang *et al.* [17]	0.7091	0.05	0.9189	our method vs Jiang *et al.*	13.5	−16.8	2.8
Perez *et al.* [27]	0.7927	0.0779	0.9245	our method vs Perez *et al.*	1.5	−46.60	2.2
Staal *et al.* [5]	0.7775	0.0287	0.9458	our method vs Staal *et al.*	3.5	60.28	−0.1
Zana *et al.* [20]	0.765	0.0266	0.9448	our method vs Zana *et al.*	5.2	56.39	−0.04
2nd observer	0.7967	0.0278	0.9497	our method vs 2nd observer	1.0	49.64	−0.6

Comparison of performance between the recent studies according to the second class of the images, including 1th, 5th, 11th, 15th, 16th, 19th test images from the DRIVE database.

**Table 4:** Quantitative evaluation of vessel segmentation algorithms related to the third class of the images.

Method	TPR	FPR	ACC	Improvement ( 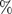 )	TPR	FPR	ACC
Our method	0.7636	0.0348	0.9478				
Jiang *et al.* [17]	0.6182	0.0306	0.9245	our method via Jiang *et al.*	24	13.73	2.5
Perez *et al.* [27]	0.7229	0.0508	0.9202	our method via Perez *et al.*	5.5	−31.5	3.0
Staal *et al.* [5]	0.6952	0.0211	0.9427	our method via Staal *et al.*	9.8	64.93	0.6
Zana *et al.* [20]	0.5915	0.0152	0.9343	our method via Zana *et al.*	29.1	128.9	1.4
2nd observer	0.7118	0.0202	0.9466	our method via 2nd observer	7.3	72.28	0.1

Comparison of performance between the recent studies according to the second class of the images, including 2th, 3th, 4th, 7th, 8th, 10th, 13th, 14th test images from the DRIVE database.

**Table 5:** Quantitative evaluation of vessel segmentation algorithms related to the 20 images from the test set.

Method	TPR	FPR	ACC	Improvement ( 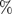 )	TPR	FPR	ACC
Our method	0.7556	0.0344	0.9475				
Jiang *et al.* [17]	0.6220	0.0318	0.9244	our method via Jiang *et al.*	21.5	8.18	2.7
Perez *et al.* [27]	0.7123	0.0524	0.9196	our method via Perez *et al.*	6.1	−34.35	3.0
Staal *et al.* [5]	0.6969	0.0214	0.9441	our method via Staal *et al.*	8.4	60.75	0.4
Zana *et al.* [20]	0.6125	0.0163	0.9372	our method via Zana *et al.*	23.4	111.04	1.1
2nd observer	0.7316	0.0208	0.9470	our method via 2nd observer	3.3	65.38	0.05

### Validation of width measurement accuracy

#### Comparison with manually detected edge images

In order to evaluate the reliability of automatic vessel edge detection including width measurements, we make use of the images included in the REVIEW database. This comprises of three Image Sets (IS) containing full fundus images: high-resolution (HRIS), central light reflex (CLRIS) and vascular disease (VDIS) with each set containing representative images that are particularly large, show visible pathologies and have vessels exhibiting prominent central light reflexes, respectively. A fourth set, the kick-point image set (KPIS), contains downsampled high-resolution images of several large diameter non-tortuous vessels. The database also offers manual width measurements made by three independent observers using a custom software tool for marking vessel edge points, so that the ground truth edge points are considered to be the average of the measurement made by the three observers at the same location in a vessel segment. A total of around 2000 locations are available for vessel width analysis.

**Figure 14:**
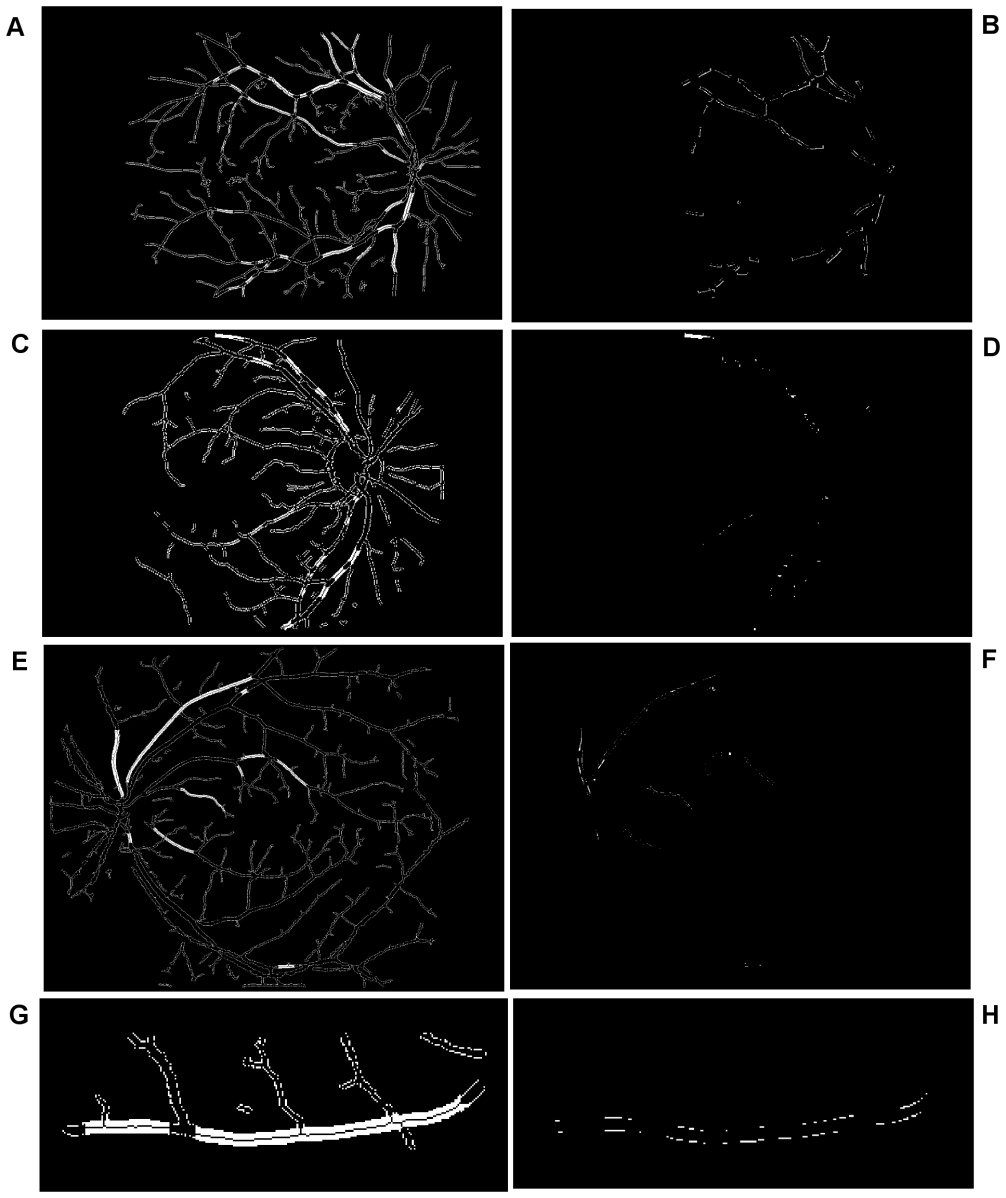
Comparison between manually detected edge images using the image datasets of HRIS, CLRIS, VDIS, and KPIS. (A), (C), (E), (G) Overlay between the vessel edge points calculated from our algorithm located on top of the manually segmented images with our centreline going through the middle part of the vessel segments. (B), (D), (F), (H) The difference between the two closed version of edge image from our algorithm and background truth, where the value of corresponding standard deviation is as: 1.11, 1.49, 1.55, 1.32.

Considering the similarity between the vessel widths in ground truth manually delineated edge points and the width measured entirely by our algorithm, width measurement accuracy cannot readily be quantified. The edge image produced by our method will differ from the manual edge detection, which will cause measurement locations and angles to not match up. One way, however, results in good agreement with the manually delineated vessels by overlaying the vessel edge points calculated from our algorithm located on top of the manually segmented images. In order to achieve the manually segmented images, we conduct a morphological close operation on the ground truth points with structuring elements of size 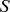 and this closed version is used as the ground truth segments. The closed versions of the manually delineated edges are obtained with variable 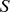, only if at this value, all edge points can be connected to form segments—this is for accurate computation and comparison. We locate the centreline on the overlay image, where the centreline is produced using morphological thinning operation on our segments for the calculation of the widths in relation to the vessels. The resultant images with such processing are illustrated in Fig. 14(A), (C), (E), (G), which correspond to the HRIS, CLRIS, VDIS, and KPIS respectively. The corresponding images used from the REVIEW database are named as: HRIS001, CLRIS002, VDIS006, KPIS001 image datasets. These images show good agreement between the edge points produced by our algorithm and the ground truth segments, with centreline (black line) within the ground truth segments, with most pixels within the middle. In order to reflect the error in width measurement between the proposed algorithm and the ground truth, the morphological closing operation is also conducted on the selected edge points from our algorithm that are matched with the edge points detected manually. The matched edge points are calculated as follows. The morphological dilation operation on the closed version of manual edge points is obtained and the dilated version is used as the mask to select the position of edge points for comparison in width measurement. The difference between the two closed version of edge image are illustrated in Fig. 14(B), (D), (F), (H).

The errors mainly arise from the following: (i) The vessel width calculated using our algorithm is 1–2 pixels wider than the ground truth, i.e. Fig. 14(B) and (D); (ii) as illustrated in Fig. 14(D), the tight border of the image is not recognised by the binarisation, which results in the vessel pixels being misclassified as background; (iii) when conducting the morphological closing operation, the closed version tends to merge minor background pixels into vessels if the edge profiles fail to possess sufficient smoothness along the edges of vessels or if a small amount of real edge points occurring in the images are missed due to our edge detection algorithm. Such effects are illustrated by Fig. 14(F).

#### Quantification of the performance measures

**Figure 15:**
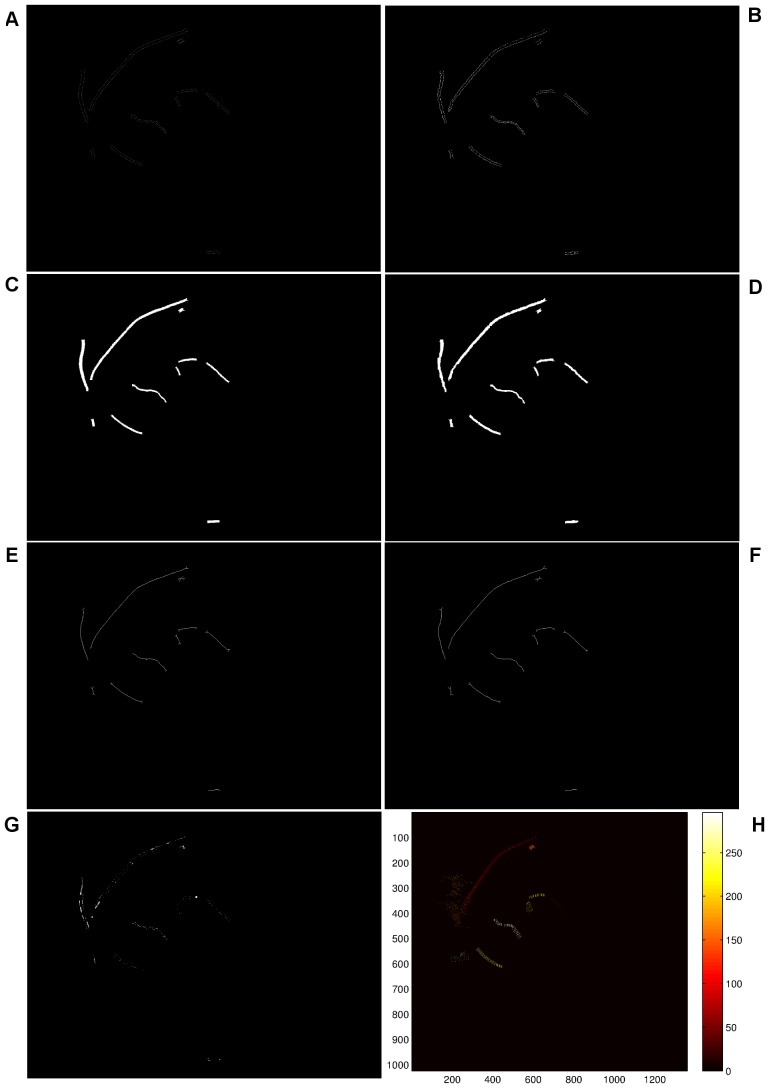
Illustration of the procedure to calculate width of vessel segment and the relevant deviation according to the VDIS image. (A) Background truth plot. (B) Detected edge via our method in the region of interest in relation to background truth. (C) and (D) Illustration of segmentation of (A) and (B). (E) and (F) Illustration of centreline of (C) and (D). (G) The error image of difference between (C) and (D). (H) Colour coded lines between each pair of ground truth coordinates.

**Figure 16:**
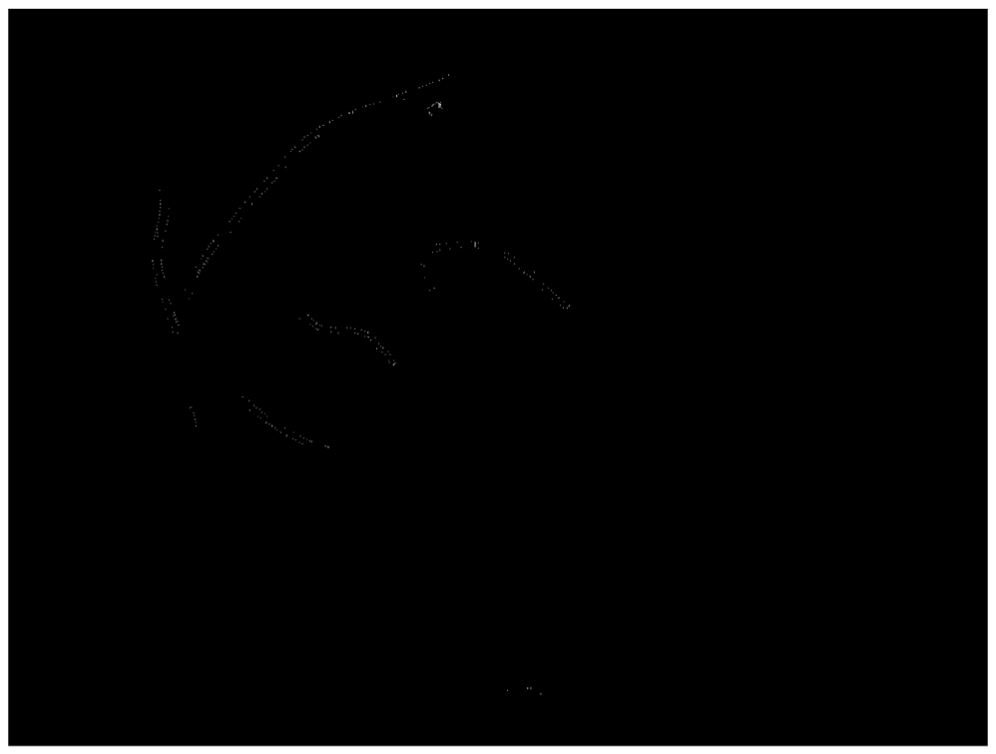
Extraction of the error image in the region that the error image is overlapped with the straight line based image of Fig. 15(H).

In order to qualify the comparison of the images mentioned above, we select successful measurement percentages (labelled by 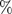), mean vessel widths (labelled by 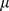) and standard deviations of the measurement error 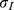. A successful measurement percentage means that each ground truth centre point should be associated with the closest detected centre point where the distance between both points is less than or equal to the true vessel width at that location. When determining comparable measurements for our algorithm, we keep the association only if the centreline calculated using the morphological thinning operation goes inside the vessel segments from ground truth data without running outside. A reduction in the measurement success percentage in these cases may indicate that the vessel is not detected. To quantify the measures of mean vessel widths, the points afforded by ground truth are used. For our algorithm, instead of computing the Euclidean distance between each pair of points from detected edges, the number of pixels in each segment, cf. Fig. 15(C) and (D), is calculated, which is then divided by the number of the pixels of the associated centreline, i.e. Fig. 15(E) and (F). The segments are obtained via morphological closing operations on edge detected images, cf. Fig. 15(A) and (B), and the centrelines are archived afterwards via morphological thinning operations on these segments. The performance of measured errors is evaluated by considering the standard deviation of errors. The errors in an image are defined as the difference between the morphological closed version of detected edge points and ground truth points at the same position of the vessels. In order to obtain the same position, straight lines are drawn that go through each pair of coordinates produced by averaged human observations Fig. 15(H). The color-coded image derived from each pair of coordinates of background truth. The error image, i.e. Fig. 15(G), is the difference between the our segment image cf. Fig. 15(D) and background segmentation, i.e. Fig. 15(C). We calculate the number of pixels in the region, i.e. shown in Fig.16, that the error image, i.e. Fig. 15(G), overlaps with the image of straight lines, i.e. shown in Fig. 15(H). The number of error pixels are then squared and summed up, and the total number of lines, shown in Fig. 15(H) are used to calculate the standard deviation. The method to qualify images for the performance measurement avoids such an issue that ground truth points cannot be uniquely matched with detected points when the detection is successful. This is due to different size of space existing in the ground truth data [11].

The performance of the proposed edge detection method is evaluated based on four retina images, related to the image sets: HRIS, CLRIS, VDIS, KPIS. The HRIS image sets are downsampled by a factor of four before being input into the test algorithms, and it is these downsampled measurements that are reported in the REVIEW database. Since manual measurements are made on the original images, vessel widths are considered to be known to an accuracy of 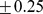 pixels [35]. Table 6 presents the performance measurements on REVIEW database. The vessel width measurements obtained using the edges produced by our method are compared against the measurements carried out by the human observers. For comparison purposes, the relevant results according to other methods in the literature are involved in the table. These methods include: that of Gregson *et al.* [48], Graph *et al.* based method [22], 1D Gaussian (1DG) [22] and 2D Gaussian (2DG) [50]. The results listed in the Table 6 above the double line use the datasets from the REVIEW database, named as: HRIS001, CLRIS002, VDIS006, KPIS001. The results listed in the table below the double line are averaged results according to the whole REVIEW database, which lead to a slightly different representation of results, but these results can be referred to for comparison.

**Table 6:** Comparison of performance between the result of our edge detection method and ground truth data points from the REVIEW database.

	HRIS			CLRIS			VDIS			KPIS		
Method	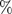	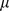	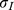	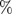	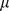	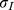	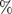	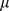	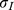	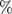	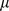	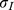
Our method	100	5.11	1.11	100	12.78	1.49	100	9.22	1.55	100	7.51	1.32
Standard	100	4.69	0.00	100	14.49	0.00	100	8.67	0.00	100	7.54	0.00
O1	100	4.58	0.34	100	14.44	0.52	100	8.65	0.52	100	7.95	0.45
O2	100	4.68	0.29	100	14.50	0.69	100	9.01	0.69	100	7.56	0.31
O3	100	4.80	0.27	100	13.92	0.54	100	8.48	0.54	100	7.61	0.33
Gregson	100	7.64	1.48	100	12.80	2.84	100	10.07	1.49	100	7.29	0.60
1DG	99.6	3.81	0.90	98.6	6.30	4.14	99.9	5.78	2.11	100	4.95	0.40
2DG	98.9	4.18	0.70	26.7	7.00	6.02	77.2	6.59	1.33	100	5.87	0.34
Graph	100	4.56	0.57	94.1	14.05	1.78	96.0	8.35	1.43	99.4	6.38	0.67

In this Table, successful measurement percentages is labeled by 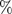, mean vessel widths is labeled by 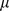, and standard deviation of the measurement error is labeled 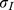. The vessel width measurements obtained using the edges produced by our method are compared against the measurements carried out by the human observers and methods in literature. These literature methods include: that of Gregson *et al.* [48], Graph *et al.* based method [49], 1D Gaussian (1DG) [22] and 2D Gaussian (2DG) [50]. The results listed in the table above the double line use the datasets from the REVIEW database, named as: HRIS001, CLRIS002, VDIS006, KPIS001. The results listed in the table below the double line are averaged results according to the whole REVIEW database, which lead to slightly different representation of results, but these results can be referred for comparison.

## Discussion

The results reported in Tables 2–5 show that, the values related to true positive rate calculated using our algorithm exceeds recently published results (increased from 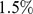 to 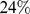), and are comparable to the performance of human observers (increased up to 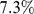). This is the most distinct improvement of our algorithm. In addition, the averaged accuracy calculated using our algorithm to process the third class of images outperforms previous methods (increased from 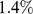 to 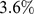), illustrated in Table 4. That is to say that the combination operation of our method is especially effective in dealing with complex cases, where both non-uniform illumination and pathological tissues are present. The average accuracy of our segmentation approach in relation to the first two classes of the images, reported in Tables 2 and 3, is slightly weaker but comparable to the methods related to the work carried out by Staal *et al.* (increased 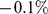) and Zana *et al.* (increased 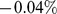). This is mainly because no more post-processing is used to remove the possible noise from background, and the interim binarisation steps tends to enlarge effects of noise when using it to eliminate the central light reflex. Our method performs significantly better than the recently reported algorithms. As shown in Table 5, our performance measures for both pathological and normal images are higher (increased from 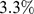 to 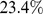) than those achieved by the other authors methods. Though our method shows increased errors generated in the misclassification of retina vessels, i.e. false positive rate when compared with the method reported by Zana *et al.* (increased 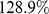 when processing Class 3 images and 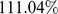 when processing all images), the averaged classification accuracies are still 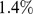 and 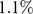. The reason for the increased false positive rate has been discussed in the previous section, with the illustrations in Fig. 11 and Fig. 12. The main types of errors in relation to the true positive rate come from partially or completely missing thin vessel branches. The expected consequence is produced mainly in thin low-contrasted vessels. It is normally related to curvature detection, which is used for localisation of blood vessels but unable to generate significant responses in regions with weakened intensity transition. The need to discriminate between valid segments and background noise prevent the reconstruction of some vessel areas.

For reproducibility, the relevant parameters used by our segmentation algorithm are: Structure elements to produce top-hat preprocessing: disk shaped with radii from 10 to 60; the structure element used for morphological H-minima transform to achieve texture mapping: disk shaped with radii from 4 (only for 08_test image) or 8 (all the remaining images from the test sets); intensity threshold value to produce mask using binarisation with threshold chosen for entropy maximisation: from 0.3 to 0.8; intensity threshold value to produce entropy filtering regarding large vessels with central light reflex: from 0.8 to 3; Alternatively, we also suggest to use gray level of colour image, instead of green channel image, to achieve entropy filtered vessel segmentation in the relevant large vessel regions; the threshold used for detection vessel intensity (larger than): from 0.04 to 0.2; the connectivity constraint (larger than): from 5 to 16.

The results presented in Table 6 are the quantification of performance in relation to our edge detection algorithm. All of the edge profiles detected by our edge detection algorithm are successful. Different from traditional evaluation of edge images for width measurements, we propose the evaluation method according to the number of pixels shown in a segment image where the edges are morphologically closed to avoid the mismatch in position of each pairs of edge pixels. The mean vessel width estimates more consistently close to the ground truth, with difference around 1 to 2 pixels or so. The average of standard deviation is comparable with methods in literature, with slightly large compared to ground truth. The reasons of the errors occurred have been discussed and illustrated in Fig. 14(B), (D), (F), (H), mainly from the misclassified pixels as discussed before.

## Conclusion

As distinct from multiscale detection algorithms, which are designed for specific range of vessel sizes, our proposed combined approach for retinal image segmentation adaptively explores local intensity characteristics and local vessel width information via conducting colour coded texture mapping. Two types of feature textures are investigated to identify noise regions with different size. A major feature of the method is its adaptability to particular image intensity properties with different noise contents. In addition, the algorithm described here automates the analysis of retinal vessel widths. It allows the fast calculation of vessel widths all along the length of each vessel rather than at specific points of interest. The quantitative performance results of both segmentation and width measurement show that our method effectively detects the blood vessels with average accuracy of above 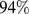, average TPR of 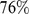, average FPR of 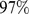, and the blood vessel width with size of 5.11, 12.78, 9.22 and 7.51 (in pixels) related to HRIS, CLRIS, VDIS and KPIS images, respectively.
